# Recent Advancements in Wearable Hydration-Monitoring Technologies: Scoping Review of Sensors, Trends, and Future Directions

**DOI:** 10.2196/60569

**Published:** 2025-06-13

**Authors:** Nazim A. Belabbaci, Raphael Anaadumba, Mohammad Arif Ul Alam

**Affiliations:** 1 Miner School of Computer & Information Sciences University of Massachusetts Lowell Lowell, MA United States; 2 National Institute on Aging NIH Bethesda, MD United States; 3 UMass Chan Medical School Worcester County, MA United States

**Keywords:** wearable technology, hydration monitoring, sensors, trends, artificial intelligence

## Abstract

**Background:**

Monitoring hydration is crucial for maintaining health and preventing dehydration. Despite the potential of wearable devices for continuous hydration monitoring, health research hasn’t fully explored this application, and clear design guidelines are absent. This scoping review aimed to address this gap by analyzing current research trends and assessing the potential impact of wearable technologies for hydration monitoring.

**Objective:**

This review comprehensively examined recent advancements in wearable hydration-monitoring technologies, focusing on their capabilities, limitations, and research and prototype designs. It explored various sensors and technologies used to track hydration, compared their advantages and disadvantages, identified trends in wearable hydration-monitoring devices, evaluated their accuracy and reliability against established benchmarks, and reviewed commercially available products to bridge research findings and practical applications.

**Methods:**

Following the PRISMA-ScR (Preferred Reporting Items for Systematic Reviews and Meta-Analyses extension for Scoping Reviews) guidelines, we systematically searched PubMed, IEEE Xplore, and Google Scholar for studies (2014-2024) on noninvasive wearable devices that used physiological biomarkers. Validation with human participants or comparisons with gold standards was required. Data extraction covered study characteristics, sensor technologies, validation methods, and commercial product analysis. In addition to academic research papers, gray literature was included through a Google Scholar search to investigate commercial products in the field of hydration monitoring. This approach ensured a broader perspective on technological advancements and market trends.

**Results:**

The review synthesized 63 articles selected from 156 included for full-text analysis. The literature was categorized based on sensor types, including electrical, optical, thermal, microwave, and multimodal sensors. Most studies (47/63, 75%) examined the effects of hydration on physiological parameters, with some (16/63, 25%) focusing on hydration status during physical activity or in specific environmental conditions. Commercially available products from 8 companies were also evaluated for their technological features, functionalities, and applications. The dominance of electrical sensors in research was highlighted due to their ease of use and integration into wearable devices. While fewer in number, optical sensors demonstrated higher precision and provided molecular-level insights. The emergence of multimodal sensors suggests a trend toward combining technologies to improve accuracy, as reflected by their increasing publication share. Other sensors, such as thermal and microwave-based sensors, occupied specialized niches. The growing acceptance of optical-based wearables in the market reflects their cost-to-precision effectiveness.

**Conclusions:**

Wearable hydration-monitoring devices provide real-time assessments of hydration status, but challenges remain in ensuring their reliability, accuracy, and applicability across diverse populations and conditions. Future directions for research include standardized protocols, extensive clinical trials, sensor miniaturization, and enhanced wearability. Multimodal systems that integrate various sensors with artificial intelligence–driven analysis hold promise for personalized hydration management. This review offers detailed insights into the strengths and challenges of sensor technologies, paving the way for practical skin hydration-monitoring solutions.

## Introduction

### Background

Water is a fundamental component of the human body, accounting for 75% of body weight in infants and 55% in older adults [1]. It plays a crucial role in the functioning of various organs. Maintaining proper hydration is essential for human health as it ensures that the body’s physiological processes function optimally. When dehydration occurs, a complex array of physiological mechanisms is triggered to preserve homeostasis. Dehydration reduces blood volume and increases plasma osmolality, which stimulates the release of hormones such as antidiuretic hormone and aldosterone. These hormones work to retain water and sodium, aiming to restore fluid balance. However, these compensatory mechanisms, while crucial, can only temporarily sustain function. Prolonged or severe dehydration can lead to impaired cellular function, disrupted thermoregulation, and compromised cardiovascular stability [2].

Conversely, overhydration disrupts the body’s electrolyte balance, most notably causing hyponatremia, a condition characterized by dangerously low sodium levels in the blood. This imbalance can lead to neurological symptoms, including confusion; seizures; and, in extreme cases, brain swelling. Specific medical conditions such as hydrocephalus can further complicate the effects of overhydration, necessitating careful management of fluid intake to prevent exacerbation of intracranial pressure [3]. The negative impacts of dehydration are especially pronounced in populations at elevated risk, such as athletes who lose significant amounts of fluid during intense physical activity and older individuals whose ability to sense thirst may diminish with age. On the other hand, overhydration, though less common, also presents significant health risks, particularly for those with underlying conditions such as heart failure or kidney disease. These populations are vulnerable to both dehydration and overhydration, making accurate and timely monitoring essential [2,4]. Understanding these biophysical and biochemical responses to hydration status is key to designing effective monitoring systems. The sensors discussed in this review are designed to detect changes in hydration by leveraging these underlying physiological mechanisms, offering real-time insights into an individual’s hydration status.

Given the complexities and the urgent need for noninvasive, real-time hydration-monitoring systems, as highlighted by numerous studies [5], recent advancements in and the widespread adoption of wearable technology present a promising solution. Over the past few years, not only have wearables become integral to daily life for many [6-9], but their potential for scientific data collection and evaluation has also significantly increased. This is especially evident in health and fitness monitoring, where wearables are being used for various metrics [9-11].

In the realm of wearable technology, a diverse range of devices, from sophisticated smartwatches [12,13] to innovative smart clothing [14], is revolutionizing how we monitor health parameters. One particularly notable advancement is their ability to monitor hydration levels, an often overlooked but essential aspect of health. These wearables use cutting-edge methods such as analyzing electrolyte variations in sweat or using electrical and optical sensors for cellular-level hydration assessments. This technique exploits the optical properties of tissues to provide a more nuanced understanding of hydration status.

The implications of these technological strides are profound. For the first time, continuous, noninvasive hydration monitoring is within reach, offering a potential safeguard for at-risk populations. This marks a new era in preventive health care, where the risks associated with hydration can be proactively mitigated, thereby enhancing the quality of life. However, this promising future is not without its challenges. Recent research in hydration monitoring, while encouraging, reveals significant gaps. A major shortfall is the lack of robust clinical studies, particularly those focused on specific diseases or clinical conditions [15-17]. This limitation raises questions about the applicability and accuracy of these technologies in real-world scenarios as the absence of targeted research hinders our understanding of their effectiveness in addressing particular medical challenges. Furthermore, most wearable technologies are still in their developmental phase [17-19], creating uncertainty about their reliability and effectiveness in practical applications. In addition, the sample sizes in existing experiments often lack statistical significance, primarily due to challenges in recruiting participants and the logistical complexities of monitoring hydration in real-world settings. These limitations diminish the reliability of the findings, making it difficult to draw definitive conclusions. Moreover, much of the research is narrowly focused on specific population groups [20], which may not fully represent the diverse needs of the broader population. This lack of inclusivity in research design could skew the results and limit the universal applicability of wearable technologies.

### Objectives

In this review, we aimed to fill a notable gap in the current literature by providing an in-depth exploration of the latest developments in wearable and portable hydration‑tracking solutions. We focused on populations at elevated risk of dehydration, such as athletes, military personnel operating in extreme environments, individuals involved in infant and maternal health, and older adults—and detailed their particular susceptibility [21] to dehydration given that body weight losses exceeding 2% can lead to heart‑related injury [22,23]. We will delve into the medical and operational causes and consequences of dehydration in these groups, examining both established and emerging monitoring techniques. This analysis is informed by recent advancements in wireless body sensor networks, as highlighted in studies such as those by Hao and Foster [24] and Hooper et al [25], and pays special attention to technologies that enable real-time monitoring, emphasizing their critical role in timely health intervention and preventive care. The rationale behind this scoping review was to comprehensively explore the landscape of wearable hydration-monitoring technologies, addressing key questions to enhance understanding and inform future research and development. Specifically, the objectives were as follows: (1) to examine the various types of sensors and technologies used for hydration monitoring, providing insights into their functionalities, advantages, and limitations; (2) to assess the trade-offs between different monitoring methods, highlighting the optimal use cases for each sensor type and category; (3) to identify trends and emerging techniques in wearable hydration monitoring, offering a forward-looking view of technology adoption and innovation; (4) to evaluate the precision and reliability of wearable devices compared to gold-standard methods, examining their performance across diverse populations and scenarios; and (5) to map commercial products in the market, analyzing their technological features, functionalities, and practical applications, bridging the gap between academic research and real-world implementations.

## Methods

### Overview

This review concentrated on wearable or portable devices, seeking to provide a detailed overview of recent advancements in hydration-monitoring technologies, including their capabilities and features. In addition, it aimed to assess the overall direction of development, research, and prototype design in this area. This scoping review followed the PRISMA (Preferred Reporting Items for Systematic Reviews and Meta-Analyses) and PRISMA-ScR (Preferred Reporting Items for Systematic Reviews and Meta-Analyses extension for Scoping Reviews) guidelines [26].

### Eligibility Criteria

We focused on studies from 2014 to 2024 to capture the latest advancements in wearable hydration monitoring. The past decade has seen rapid progress in sensor miniaturization, real-time physiological monitoring, and artificial intelligence (AI)–driven analytics, making earlier studies less relevant to current technological capabilities. Studies were included if they met the following criteria: (1) focus on wearable or portable hydration-monitoring systems to assess real-world usability; (2) use of noninvasive or minimally invasive physiological biomarkers to ensure practical application; (3) direct addressing of body hydration, targeting biomedical applications; and (4) reliance on physiological biomarkers rather than methods such as body motion tracking or monitoring fluid intake or extraction through smart bottles or toilets as they do not assess direct hydration status.

Exclusion criteria included studies without human participant validation or relevant performance evaluations, ensuring the reliability of the findings. Other exclusion criteria included research not published in peer-reviewed journals or reputable conference proceedings to maintain study quality and non–English-language publications due to resource constraints and replication potential.

### Information Sources and Search

The last literature search was conducted in January 2024. The search strategies were drafted and further refined through team discussion. The search incorporated keyword variations such as “hydration monitoring” and “wearable devices,” used advanced search functions in PubMed and IEEE Xplore’s Title and Abstract fields, and further extended to Google Scholar to ensure comprehensive literature coverage. The final search string—“(hydration monitoring OR hydration sensor) AND (wearable OR device)”—targeted literature published after 2014 and yielded the most complete results. Additional studies were identified through reference tracking of relevant papers to cover any potential missed papers. Other terms did not produce relevant results in either database and, therefore, were excluded. The final search string was designed to be broad and inclusive, allowing for the identification of numerous articles considered for inclusion in this study. The results duplicates were removed and then exported into a Mendeley library (Elsevier).

### Selection of Sources of Evidence

A total of 551 articles were identified initially; PubMed returned 408 (74%) articles, IEEE Xplore returned 107 (19.4%) articles, and articles from additional sources were also included (n=37, 6.7% papers that were identified from Google Scholar search and the references of the included papers that did not appear in the initial search results). After duplicate records (5/551, 0.9%) were identified and removed using Mendeley’s automated duplicate detection, followed by manual verification to resolve mismatches across databases, of the 551 initial articles, 546 (99.1%) remained. Screening based on titles and abstracts led to the inclusion of 156 studies for full-text analysis. To ensure a rigorous selection process, 2 reviewers screened the titles and abstracts of all studies for relevance. To enhance consistency, all reviewers initially screened the same set of publications, discussed the results, and refined the screening and data extraction criteria before beginning the full review. Studies that met the inclusion criteria proceeded to full-text review, conducted sequentially by 2 reviewers working in pairs.

If a study’s title or abstract did not provide enough information to determine eligibility, particularly regarding the inclusion of validation procedures, a thorough examination of the full text was conducted. Disagreements on study selection and data extraction were resolved through discussion and consensus. When necessary, a third reviewer provided a final review to resolve conflicts and ensure consistency in the selection process.

### Data Extraction and Charting Process

A data extraction and charting process was jointly developed by 2 reviewers to determine which variables to extract, including study characteristics, device specifications, sensor technology, validation methods, performance metrics, key findings, and limitations. Using these variables, structured summaries were extracted to synthesize the evidence and effectively answer the research questions and objectives. The 2 reviewers conducted data extraction using Mendeley, annotating each paper and summarizing key extracted data. To maintain consistency, a calibration exercise was conducted at the beginning of the extraction process where all reviewers screened a sample of publications, discussed discrepancies, and refined data extraction criteria. The charting form was continuously refined through an iterative process, with regular discussions among reviewers to update variables and improve clarity.

### Commercial Product Search

In addition to academic research papers, we conducted a Google Scholar search to investigate commercial products in the field of hydration monitoring. We used keyword variations from the database searches, such as “hydration monitoring wearable device” and “hydration sensor technology.” Through online research, we identified several companies. Detailed evaluations were undertaken to compare the technological features, functionalities, and applications of these commercial products. This analysis offered valuable insights into practical implementations and advancements in the field, bridging the gap between academic research and real-world applications.

In our commercial product search, we differentiated between products designed for medical purposes and those intended for general hydration monitoring. Medical-grade devices were identified based on criteria such as Food and Drug Administration (FDA) approval, clinical validation, and their use in medical settings for patient care. These products are typically characterized by higher precision, sensitivity, and adherence to regulatory standards. In contrast, products aimed at general hydration monitoring are often marketed for consumer use, such as fitness and wellness tracking, and may not have undergone the same rigorous testing or obtained the necessary certifications for medical applications. This distinction was crucial in our analysis as it directly impacted the reliability and potential use cases of the devices evaluated. Specifically, we identified and compared 8 companies based on factors such as validation, FDA approval, and technology used.

## Results

### Overview

**Figure 1:**
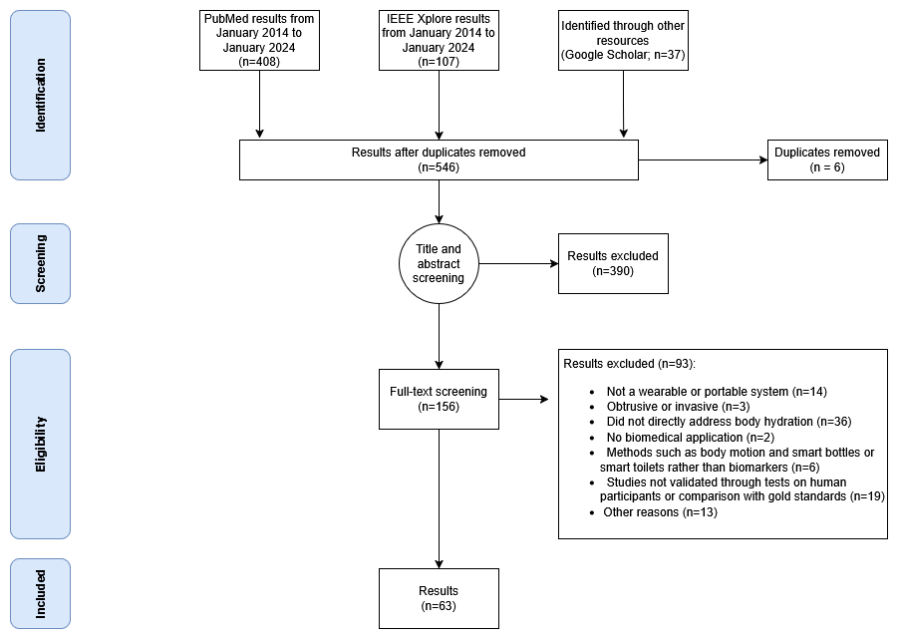
PRISMA (Preferred Reporting Items for Systematic Reviews and Meta-Analyses) flow diagram of the selection of studies on wearable and portable hydration-monitoring systems.

During the initial screening process, 546 articles were identified. After applying the inclusion and exclusion criteria, of the 546 articles, 156 (28.6%) were selected for full-text analysis. Following a thorough review, 63 articles were retained for synthesis and analysis (Figure 1).

**Figure 2:**
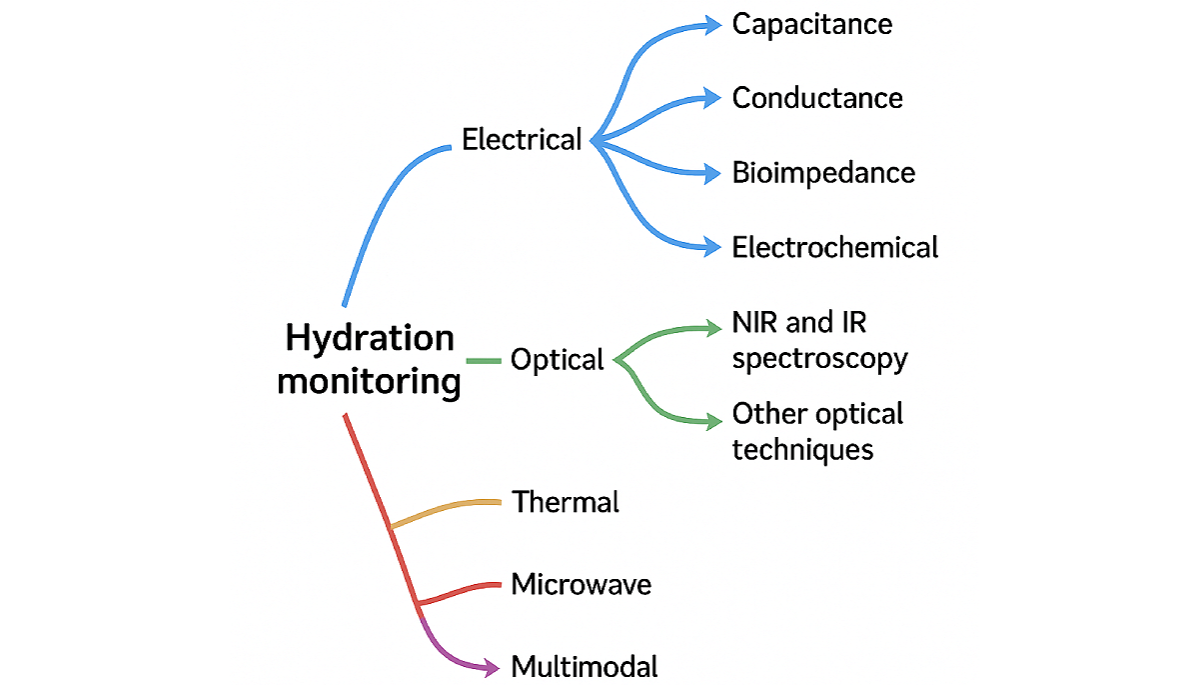
Hydration-monitoring systems categorized based on the type of sensors used. IR: infrared; NIR: near infrared.

To organize the selected literature and facilitate a comprehensive analysis, a classification taxonomy was developed based on the types of sensors used for hydration monitoring in wearable technologies. This taxonomy categorized the papers into the following groups: electrical sensors, optical sensors, thermal sensors, microwave sensors, multimodal sensors, and commercial products (Figure 2). This classification enabled a structured review of the literature, highlighting the different sensor technologies used in hydration-monitoring devices.

### Electrical-Based Sensors

#### Overview

Electrical-based sensors for hydration monitoring use various techniques to measure changes in the electrical properties of the skin. They leverage the body’s physiological changes in response to fluid imbalance. These sensors measure the electrical properties of the skin and underlying tissues, which vary according to the body’s hydration state. The main methods used include capacitance, conductance, bioelectrical impedance analysis (BIA), and electrochemical analysis, each with its unique principles, advantages, and challenges.

#### Capacitance Method

This category includes sensors that monitor hydration by evaluating the skin’s capacitance. Capacitance sensors measure the skin’s ability to store an electrical charge, which fluctuates with hydration levels. When the body is hydrated, the water content in the skin is higher, increasing its dielectric constant and capacitance. As dehydration occurs, the water content decreases, lowering the skin’s capacitance, as illustrated in Figure S1 in Multimedia Appendix 1 [16,27-30].

Yao et al [31] introduced a wearable skin hydration sensor (SHS) featuring silver nanowire electrodes. This sensor, with its flexible and stretchable nature, maintains consistent performance despite changes in humidity. It was calibrated against the MoistureMeterD commercial skin hydration measurement system [32] and packaged into a flexible wristband with an ultralow-power microcontroller unit (MCU) and Bluetooth capability. Future research should assess its adaptability to different skin layers and further develop its potential for widespread use.

Another study [33] presented a “skin hydration sensor patch” that integrates capacitive measurement with near-field communication (NFC) data transmission to smartphones. The patch demonstrated a high correlation with Corneometer results on various skin sites. A subsequent study with moderately dry skin in the face and forearm suggested upgrading the sensor probe calibration to enhance sensitivity. Future directions could include correlating changes in facial skin signs with varying hydration levels using AI-driven systems that analyze selfies.

In another study [34], capacitive textile sensors were developed for continuous skin hydration monitoring, with the goal of integrating them into everyday clothing. This study emphasized the sensors’ potential for real-time health assessment and improved patient comfort. However, challenges related to sensor absorption, long-term performance, and measurement pressure were noted, suggesting that future research should address these issues to enhance the reliability and applicability of textile sensors.

#### Conductance Method

This section explores papers that focused on hydration monitoring through sensors that measure skin conductance. Conductance-based hydration monitoring assesses skin moisture levels by measuring the ease of electrical current flow through the skin, with higher conductance indicating greater hydration.

Clarys et al [35] conducted a comparative analysis to quantify the differences between conductance-based and capacitance-based methods for hydration assessment. They examined 2 instruments, the Corneometer CM 825 (capacitance based) and the Skicon-200 EX (conductance based). Their in vitro and in vivo evaluations revealed that conductance measurements are influenced by electrolytes, whereas capacitance measurements are not. Capacitance allows for probing deeper skin layers (up to 45 µm) compared to conductance (up to 15 µm), and both methods showed strong correlations between water content and measurement accuracy.

Lu et al [15] introduced a portable device for diagnosing dehydration and its potential link to chronic kidney disease through saliva conductivity analysis. The device, consisting of an MCU, an analogue-to-digital converter, and a liquid crystal display, measures the electrical signals from saliva samples. Miniaturized electrodes reduce the sample contact area and the amount of saliva required for testing. However, significant variations in saliva conductivity due to factors such as diet, hydration status, and individual physiology present challenges to the device’s accuracy and reliability.

Rizwan et al [16] proposed using galvanic skin response (GSR) as a noninvasive marker for hydration, collecting and analyzing GSR data from individuals in different hydration states and body postures (Figure S2 in Multimedia Appendix 1 [16,27-30]). The study demonstrated advancements in noninvasive hydration monitoring but suggested that further research is needed to refine algorithms and address variability in GSR measurements.

Madhvapathy et al [36] presented a versatile and cost-effective patch based on flexible printed circuit boards (PCBs) for skin hydration monitoring and dermatological diagnosis. The platform uses a thermistor to measure skin conductivity and diffusivity, enabling the measurement of volumetric water content up to approximately 1 mm in depth. The sensor communicates wirelessly with smartphones using NFC technology. However, limitations include insufficient knowledge about epidermis thickness, which cannot be directly inferred from thermal conductivity data.

Wang et al [37] introduced a wearable sweat sensor platform designed for the simultaneous measurement of sweat rate and total electrolyte concentration without calibration. The platform features a fluidic controlled design that minimizes dilution effects and detects sweat. Practical tests demonstrated its capability to measure regional sweat rate, sweat loss, and electrolyte concentration, particularly for high-sweat situations such as sports. However, the platform may face challenges with low flow rates, impacting real-time detection and introducing potential bias. Addressing this limitation could improve its accuracy and applicability in scenarios with varying sweat flow rates.

Responding to limitations in existing hydration-tracking methods, Liaqat et al [38] developed a novel system based on machine learning and deep learning techniques to accurately estimate hydration levels using GSR data. This hybrid model improved accuracy, making it valuable for applications in sports, health, and well-being.

#### BIA Sensors

BIA sensors differ from conductance-based sensors by measuring impedance, which includes both conductance and capacitance, as illustrated in Figure S3 in Multimedia Appendix 1 [16,27-30]. The working principle involves an initial rapid impedance change caused by electrolyte solution filling skin voids, followed by a gradual change associated with water gradient equalization and structural changes in the skin barrier [39].

Chua [40] introduced the i-Health Watch, an integrated wearable device for continuous monitoring of heart rate (HR), hydration level, and blood glucose concentration using bioimpedance. The compact prototype incorporates 2 pairs of silver electrodes with conductive gel, enabling real-time tracking. The watch’s display provides continuous readings and issues on-screen alerts for abnormalities. The novelty lies in a parallel signal processing approach, deriving 3 physiological readings from a single bioimpedance feed, which streamlines the device. Initial testing on a single human participant demonstrated the potential for future integration into a smart telemonitoring health system.

Agcayazi et al [41] designed a wearable bioimpedance analyzer for infant hydration monitoring. The system uses an Analog Devices AD5933 impedance analyzer combined with an RFduino for Bluetooth communication with a smartphone. Validation on 2 adult participants using electrodes placed on specific body parts demonstrated a relative decrease in bioimpedance and an increase in hydration as participants consumed water progressively. However, the system requires initial calibration based on an individual’s height and weight, which may limit its applicability in certain scenarios.

Yang and Rosa [42] introduced a small, batteryless wearable device for assessing skin hydration levels. The device combines sunlight exposure computation with skin impedance and temperature measurement. The goal was to incorporate it into a bracelet or ring for simultaneous UV index, skin hydration, and temperature measurement, which current UV trackers do not offer. Despite its simplicity, affordability, and energy efficiency, the device proves useful for detecting skin impedance variations due to water loss from sunlight exposure. Testing during the summer is crucial to establish correlations between skin impedance changes and higher UV indexes.

Sunny et al [43] developed a cost-effective bioimpedance sensor that uses 4 gel electrodes on the skin surface to mimic skin properties and detect hydration changes. Accuracy was validated across a range of skin tones and types. The sensor shows promise for detecting hydration changes in both phantom and human skin experiments. Future research could focus on enhancing the sensor’s performance and usability in portable or wearable modes and standardizing experimental setups.

Leonov et al [44] introduced a sensor with significant sensitivity to hydration changes, detecting sweat loss with a limit of 55 mL (approximately 1.86 oz) in the worst-case scenario. Although the device showed sensitivity to hydration changes of approximately 700 mL (approximately 23.67 oz), more refined protocols and device improvements are necessary to enhance reliability for practical daily-life applications.

Matsukawa et al [27] presented a method for continuous monitoring of skin hydration using nanomesh electrodes attached to the left ventral forearm. These electrodes adhere comfortably to the skin and allow for water vapor permeability during extended wear, enabling impedance measurements without pressure application. Impedance results correlated with hydration levels measured using a Corneometer. However, intense sweating may cause short-circuiting between electrodes due to the lack of an encapsulation layer. The research plan addresses this challenge by adding an encapsulation layer.

Veeralingam et al [45] developed a novel “body hydration analysis system” to enhance running performance by assessing skin hydration levels through impedance variations. The system uses the open-source QueSSence board and an AI-driven k-nearest neighbor algorithm for efficient data analysis. On the basis of a semiconductor compound with high electronic properties, the sensor demonstrated high conductivity, ideal for wearable applications. However, further validation and integration of these technologies into user-friendly wearable devices for real-time monitoring during athletic activities are necessary.

Valentin et al [21] created a wearable MCU-based BIA device for precise hydration measurements with low power consumption. Electrodes were placed below the right shoulder blade and on the bottom left of the abdomen. After an initial measurement, participants consumed 200 mL (approximately 6.76 oz) of water, and a second measurement was taken after 15 minutes. The experiment resulted in an impedance recording accuracy of <2%, suggesting potential for future clinical applications. Further research should address limitations such as sample size and long-term monitoring to validate the method for clinical use.

Songkakul et al [18] introduced a miniaturized wearable bioimpedance spectroscopy system for continuous tissue hydration monitoring, focusing on electrode optimization and advanced data processing. The system comprises Bluetooth connectivity and an analogue front-end circuit integrated with conformable, flexible, and stretchable silver nanowire electrodes. The system operates with a lithium battery providing 18 hours of functionality. The 4-electrode configuration uses 2 electrodes for current source and sink, whereas voltage is measured across the other 2 electrodes. Further research is needed to validate the system in vivo and improve its accuracy and usability in real-world applications.

AlDisi et al [17] developed and tested 2 wrist-worn interdigitated electrode designs for assessing hydration levels using BIA. The study demonstrated that interdigitated electrode designs could accurately measure hydration without complex models, addressing classification challenges and impedance reading influences. The experiments involved 6 participants classified into severe dehydration, mild dehydration, and hydration states. Although promising for hydration assessment without complex calibration, further clinical studies with a larger sample size are necessary to establish robust classification criteria and address variations between body sizes and testing sessions.

Thomas et al [19] introduced a molybdenum diselenide and polyvinyl alcohol–based wearable platform for pulse rate monitoring and skin hydration sensing. The molybdenum diselenide and polyvinyl alcohol compound behaves like a combination of resistance and capacitance in parallel, with the skin acting as resistance when the sensor is applied. Experiments involving moisturizer application and changes in relative humidity demonstrated the sensor’s responsiveness and accuracy in detecting skin hydration levels. While the fabrication process is described as simple and cost-effective, it still requires specialized techniques such as hydrothermal synthesis, which may limit scalability and accessibility for widespread adoption.

Jang et al [46] presented a textile-based wearable sensor designed for stable and reliable monitoring of skin impedance changes related to hydration levels. The sensor can detect different hydration levels across various body parts while maintaining stable skin contact. Time-dependent monitoring of skin hydration on the hand demonstrated the sensor’s ability to detect changes over time, with reliable results and fewer SDs than ground-truth sensors. Future research could focus on designing more intricate electrodes to improve sensing performance or adjust for different skin colors and types.

Tonello et al [20] described a multisensory wearable that integrates flexible sensors on a bracelet-shaped substrate for temperature, body impedance, and skin hydration monitoring. The device measures total impedance from 3 sensors: a resistance temperature detector, a BIA sensor, and a hydration status sensor. This combination enables continuous, noninvasive hydration level measurement through temperature, local hydration monitoring, and global body composition analysis. Preliminary in vivo validation on 2 volunteers demonstrated the sensors’ capability to detect different hydration conditions. Future work will focus on refining sensor design and conducting reproducibility and stability tests.

SkinUp [47] measured skin moisture and oil levels using impedance, validated against the Corneometer CM 825, offering a portable solution for skin hydration measurements across various regions, including the forearm, cheeks, and forehead.

#### Electrochemical Analysis

Electrochemical analysis uses various techniques to monitor electrolyte concentrations [48]. These include sweat sensors that use ion-selective electrodes (made of field-effect transistors) with microfluidics and low-noise electronics for precise electrolyte level monitoring [49,50]. Other approaches include waterproof, epidermal microfluidic devices for capturing and analyzing sweat underwater, as well as chemiresistor-based sensors for monitoring sweat dynamics [51,52].

In their study, Culver et al [48] focused on monitoring electrolytes in sweat using a wearable sweat sensor. The objective was to evaluate the sensor during a simulated special operations field event conducted at the US Air Force Academy. While the sensor showed promise for hydration monitoring, challenges arose from limited sweat availability for detection. The study explored sensor placement on the lower back, a region known to produce more sodium-rich sweat. The device incorporates ion-selective electrodes with microfluidics and low-noise electronics to ensure precise and reliable monitoring of sodium ion and potassium ion levels in sweat [49]. Challenges include improving sensor shelf life and stability and reducing its footprint.

Reeder et al [51] introduced waterproof, epidermal microfluidic devices for capturing and analyzing sweat underwater. Designed for athletics and fitness, these devices allow for real-time monitoring of fluid loss and electrolyte concentrations in aquatic environments. Field trials have demonstrated quantitative in situ measurements of sweat chloride concentration, local sweat loss, and skin temperature during physical activity. The devices are made of soft, waterproof materials and are designed for seamless operation in extreme environments. Future research aims to explore the physiology of aquatic sweating and validate the device’s utility in various settings.

Parrilla et al [52] introduced a wearable paper-based chemiresistor for monitoring sweat dynamics, focusing on sweat rate and loss. This sensor measures volume of aqueous solution at the microliter scale by detecting resistance changes along a conductive paper substrate. The study outlined the sensor’s analytical performance, sensing mechanism, and effectiveness in monitoring sweat loss during exercise. Challenges include optimizing sensitivity and reliability across different conditions.

Lafaye et al [50] presented a real-time multi-sensing wearable platform for continuous sweat biomonitoring. One prototype uses an ion-sensitive electrode soft-sensing patch, whereas the other uses silicon-based sensors and paper microfluidics. Both platforms integrate multisensory arrays for measuring sodium, potassium, and pH in sweat. Initial results from prototypes placed on athletes during exercise have demonstrated the platform’s efficacy. Body water loss (BWL) percentage was used as a quantitative indicator of the participants’ hydration status. Machine learning algorithms predict BWL from biomarkers such as HR and sweat sodium concentration. Future work includes expanding experiments with more participants and correlating sweat sensor recordings with other biomarkers.

Yang et al [53] highlighted a cost-effective wearable sweat sensor using screen printing technology for real-time monitoring of potassium ion and sodium ion concentrations in human sweat. A 10-day continuous monitoring experiment revealed a close relationship between potassium ion and sodium ion concentrations and hydration status. The fabricated sensors demonstrated sensitivity, linearity, repeatability, resistance to interference, and mechanical deformation resistance, making them suitable for sweat-sensing applications.

#### Comparative Overview of Sensor Techniques

These various techniques for monitoring skin hydration levels each have distinct strengths and limitations. The selection of an appropriate sensor depends on the specific application and user preferences, considering factors such as precision, comfort, ease of use, and the duration of wear. Table 1 summarizes the findings of electrical-based sensors used for hydration tracking.

**Table 1:** Comparative analysis of skin hydration–monitoring techniques.

Sensor type	Strengths	Limitations	Studies
Capacitance	Extremely sensitive to skin moisture variation and simple to use	Potentially less precise in measurement	Yao et al [31] Flament et al [33] Balaban and Blecha [34]
Conductance	Balanced accuracy and user-friendly	May not be as comprehensive in assessment	Lu et al [15] Rizwan et al [16] Madhvapathy et al [36] Liaqat et al [38]
Bioimpedance	Comprehensive assessment and combines multiple metrics	Limited long-term wearability and requires continuous contact	Chua [40] Agcayazi et al [41] Sunny et al [43] Leonov et al [44] Matsukawa et al [27] Veeralingam et al [45] Valentin et al [21] AlDisi et al [17] Jang et al [46]
Electrochemical	High sensitivity and specificity and detects specific ions	Complex setup, requires careful calibration, and potential limitations in practicality	Culver et al [48] Reeder et al [51] Parrilla et al [52] Yang et al [53]

### Optical-Based Sensors

#### Overview

Typically, noninvasive biomedical measurements involve directing light of a specific wavelength onto the skin to collect data. A sensor then detects the light that is either reflected, absorbed, or refracted. These data are used to quantify biomedical information. The wavelength of the light is critical in determining the depth of its penetration into the skin when transmitting an optical signal (Figure S4 in Multimedia Appendix 1 [16,27-30]). This aspect primarily distinguishes the techniques reviewed in this section. Moreover, these methods allow researchers to assess the physiological condition of internal organs through the skin [54].

#### Near-Infrared Spectroscopy

In recent years, near-infrared spectroscopy (NIRS) has become a powerful tool for comprehensive skin hydration analysis within the optical range of 750 to 2500 nm. It is selected for its ability to capture absorption bands of water, proteins, lipids, and other skin constituents, enabling precise measurement of hydration levels.

In 2013, Qassem and Kyriacou [55] used NIRS to investigate the effects of short-term skin contact with water and subsequent moisturizer application on human skin. The study involved in vivo measurements within the range of 9,002,100 nm, highlighting variations in peak values regarding water overtone and combination bands, providing insights into distinctions between skin types and moisturizer use patterns.

Visser et al [56] developed a portable prototype using an Arduino Uno32 MCU and 2 infrared LEDs emitting light at 1300 and 1480 nm. These wavelengths were chosen for their different water absorption coefficients, with 1300 nm selected for maximum skin penetration depth and 1480 nm selected for sensitivity to water concentrations. The study noted greater variability in hydration status among adult patients than among infants, emphasizing the need for universal calibration and validation protocols. Further research is required to ensure the sensor’s effectiveness across diverse demographic groups.

In another study, Visser et al [57] investigated the use of optical sensors for assessing infant dehydration. An infrared spectrometry sensor using infrared LEDs at 1300 and 1480 nm was used to maximize skin penetration depth and sensitivity to water concentrations. Conducted on 10 infants with acute gastroenteritis, the study demonstrated promising results, showing high specificity and sensitivity of the infrared spectrometry sensor in dehydration assessment. Challenges such as infant movement artifacts and data irregularities were mitigated using supplementary algorithms and repeated measurements. A larger population sample is needed before clinical deployment.

Mamouei et al [58] developed a portable sensor for skin hydration monitoring targeting dermal water content. The multiwavelength optical sensor is designed for continuous and nonintrusive monitoring using 4 LEDs emitting light at 940, 970, 1200, and 1450 nm, 3 of which correspond to water absorption peaks in the near-infrared region. Validation involved benchmarking accuracy against a high-end broadband spectrophotometer. Future work will focus on establishing appropriate algorithms for sensor calibration in the absence of reference gravimetric data, addressing limitations, and enhancing the sensor’s accuracy and usability.

Benavides et al [59] introduced a novel wearable medical device for continuous UV exposure and hydration monitoring. The device uses infrared spectroscopy, with an LED emitting light at 940 nm and a photodiode detecting unabsorbed light to measure water absorption in the skin. This enables real-time hydration assessments through a paired mobile app. Table 2 summarizes the key biomarkers and their wavelength ranges as reported in NIRS studies.

**Table 2:** Near-infrared spectroscopy biomarkers, wavelength range, and studies.

Biomarker	Wavelength range	Studies
Water content	1300 and 1480 nm	Visser et al [56,57]
Water absorption peaks	940, 970, 1200, and 1450 nm	Mamouei et al [58]
Water content	940 nm	Benavides et al [59]
Water content and sweat	940 and 1450 nm	Volkova et al [60]

#### Other Optical-Based Techniques

Other optical sensors are increasingly being used for noninvasive hydration monitoring. These sensors use various techniques such as spectroscopy across a wider range of wavelengths and photonics to analyze biomarkers in sweat or tissue.

Curto et al [61] introduced a self-contained wearable designed for real-time analysis of pH levels in sweat. The platform incorporates a surface-mount LED (smLED) and a photodiode controlled by a LilyPad Arduino MCU. Detection occurs through the photodiode and smLED positioned above and below the sensing area, respectively. Replacing standard LEDs with smLEDs reduced the bulkiness of the system. Challenges include refining sensitivity, specificity, and accuracy, as well as expanding the range of detectable analytes.

Ozana et al [62] introduced a wearable optical sensor for noninvasive glucose concentration detection and dehydration level assessment. The sensor uses 2 optical techniques: remote vibration source extraction and polarization rotation of light. It includes 4 LEDs emitting wavelengths from 600 to 1150 nm and a green laser (532 nm), along with a camera connected to a computer in a braceletlike setup. Dehydration measurements were taken in a 50 *°*C chamber. Challenges include implementing a motion cancellation mechanism and developing a robust automatic calibration process.

Perkov et al [63] investigated optoacoustic monitoring for assessing water content in tissue. Known for its optical contrast, ultrasound resolution, and significant penetration depth, the optoacoustic technique shows promise for skin hydration assessment. The analysis was conducted across wavelengths from 1370 to 1650 nm to optimize water content assessment in skin tissues. Challenges include optimizing wavelengths to fully exploit the potential of optoacoustic monitoring. A miniaturized complementary metal oxide semiconductor spectrometer in the 650,900-nm wavelength range designed for continuous monitoring of dermal skin hydration is presented in Figure S5 in Multimedia Appendix 1 [16,27-30]. Fabricated using a monolithically integrated filter process, the spectrometer ensures cost-effectiveness, low power consumption, and mass production capability. In the same study, Perkov et al [63] also highlighted specific wavelengths, such as the 970-nm water absorption and 930-nm fat absorption peaks, showing the potential of the device for wearable skin biomarker measurements and its versatility beyond hydration to applications such as HR and subdermal markers such as glucose or lactate detection [28]. However, the study was limited by a small sample size and lacked extensive validation across diverse populations and clinical conditions.

Sandys et al [64] conducted a pilot observational study to evaluate the Sixty wearable hydration monitor in patients undergoing hemodialysis. The device uses diffuse reflectance spectroscopy and machine learning to detect subdermal fluid levels. Photonic sensors and wavelengths from 530 to 950 nm were used to determine fluid status based on reflected light. The study compared the device’s accuracy with bioimpedance measurements during dialysis sessions and overnight for 3 weeks. Successful validation could lead to integration into a comprehensive algorithm for managing fluid overload in interdialytic periods.

Bohman et al [65] presented a silicon photonics-based spectrophotometer for monitoring temperature and water content in tissue-simulating phantoms using semiconductor lasers. The spectrophotometer demonstrated its utility in tracking temperature and water content, showing excellent agreement with reference values. However, variables such as the complexity of human tissue could affect precision in real-world scenarios.

#### Summary of Optical Techniques and Biomarkers

Table 3 summarizes the various optical techniques and the biomarkers used for hydration tracking.

**Table 3:** Optical techniques and biomarkers for hydration tracking.

Biomarkers	Technique used	Studies
pH levels in sweat	smLED^a^ at visible light (300-700 nm) and LEDs within the 600-1150 nm range	Curto et al [61] Ozana et al [62]
Temporal changes in reflected speckle patterns were analyzed to assess skin water content	Optoacoustic monitoring (1370-1650 nm)	Perkov et al [63] Van Beers et al [28]
Localized PWC^b^ dermal fluid status	CMOS^c^ spectroscopy (650-900 nm)	Sandys et al [64]
Temperature and water content	Photonic sensor (530-950 nm)	Bohman et al [65]

^a^smLED: surface-mount LED.

^b^PWC: percentage of water content.

^c^CMOS: complementary metal oxide semiconductor.

### Thermal Sensors

Thermal methods for assessing hydration involve measuring the skin’s thermal properties, such as temperature and thermal transport, which can indicate hydration levels and blood flow dynamics. Madhvapathy et al [66] described the development of soft, skinlike thermal depth sensors to track hydration levels. The sensor measured the thermal properties of human skin at depths up to 6 mm (approximately 0.24 in) beneath the skin’s surface, addressing limitations of current methods confined to superficial layers in clinical environments.

Krishnan et al [67] introduced a wireless, battery-free sensor system for measurement of the skin’s thermal properties. The device comprises a wireless power-harvesting system, NFC-based data transmission, analogue signal conditioning on a flexible PCB, and a stretchable sensor with a temperature coefficient of resistance for precise temperature measurements. Human trials demonstrate continuous monitoring of skin thermal properties over a week without interruption. Challenges involve ensuring the accuracy and reliability of the data collected. Kwon et al [68] introduced a wireless, soft electronics platform designed for rapid measurements of hydration levels. Recent alternatives using thermal measurements with soft wireless devices have limitations such as a restricted operating range (1 cm) and sensitivity to environmental fluctuations. In response, this study presented innovative technologies to overcome these drawbacks, enabling high-speed, robust, automated measurements of thermal transport properties. The sensor module was controlled by a Bluetooth Low Energy (BLE) system on a chip with a smartphone interface.

Shin et al [69] presented a novel wireless and soft SHS designed for rapid and accurate diagnostics of dermatological health. The SHS integrates a BLE system on a chip within a flexible PCB. Pilot trials involving >200 patients in a dermatology clinic highlighted the practical applicability conducted at 3 skin locations (forehead, lower leg, and lower arm) using both the SHS and a commercial system (Delfin). The SHS operates without applied pressure, adheres gently to soft and curved skin regions, and provides rapid and objective measurements. Future research could improve this sensor by tackling potential limitations, including scalability, long-term reliability, and user comfort.

### Microwave-Based Sensors

Microwave-based sensors use microwave or electromagnetic signals to measure various hydration-related parameters. Butterworth et al [70] presented a wearable wristband for hydration monitoring using noncontact dielectric spectroscopy in the microwave range (2-6 GHz). This technology leveraged minute variations in wrist hydration, offering a unique approach to tracking overall hydration status. Potential areas for improvement include further validation studies involving a larger and more diverse sample of participants. The paper by Wang et al [71] presented a wearable radio frequency device for noninvasive real-time hydration monitoring, correlating the received signal strength indicator with BWL percentage. The device comprised near-field antenna sensor nodes, a radio frequency front end, and digital processing units. Challenges involve further validation and refining the accuracy and usability of the device.

Schiavoni et al [72] investigated a time-domain reflectometry (TDR)–based wearable skin hydration–sensing system, an electromagnetic technique showing accuracy, cost-effectiveness, and portability for potential medical applications. Challenges may involve ensuring reliability and connecting TDR measurements with physiological parameters.

In another study, the system by Schiavoni et al [73] combined TDR and frequency-domain data extraction in microwave reflectometry, developing calibration curves linking skin dielectric permittivity to frequency-domain responses, showing promise for real-time skin hydration monitoring. Challenges may include system validation, portability, and integration into wearables.

Besler and Fear [74] investigated microwave-based hydration assessment with fasting volunteers during Ramadan using a time-of-flight permittivity estimation technique to measure hydration changes throughout the day. Challenges include improving sensitivity and precision in detecting subtle hydration changes.

Cataldo et al [75] developed a flexible wearable for skin hydration sensing. The sensor underwent redesign for enhanced wearability, sensitivity, and patient comfort, incorporating a flexible rubber substrate with a Kapton layer. Future efforts will focus on system improvement and systematic characterization, including solutions for Bluetooth data acquisition and local processing. Their goal is to further miniaturize the system for full wearability by removing the liquid crystal display screen.

Bing et al [76] introduced a small, planar resonant loop sensor for water content monitoring based on electromagnetic resonance. Experiments on human hydration processes align well with simulations using documented skin permittivity properties. The wearable sensor, integrated on the human forearm, offers discrete and continuous measurements. It is crucial to note that the sensor’s sensitivity is influenced by dielectric property changes due to water content variations.

### Multimodal Sensors

This section explores research on multisensory systems designed to provide a comprehensive assessment of hydration levels by integrating various sensor types and combining various measurement techniques. Krishnan et al [77] introduced multimodal sensors for precise, quantitative in vivo monitoring of hydration levels in near-surface skin regions. The mathematical model they developed encompasses temperature, thermal conductivity, thermal diffusivity, volumetric heat capacity, and electrical impedance using a simple analysis algorithm. Challenges include evaluating the device’s performance in real-world scenarios with varying temperature and humidity.

Salvo et al [78] developed a wearable sensor for real-time sweat rate monitoring using the open-chamber method based on Fick’s first law of diffusion. The cylindrical chamber within a 3D-printed adapter calculated the sweat rate by measuring water vapor flow from the skin. The sensor, tested on 13 participants during a cycling test, consisted of an Arduino Pro Mini and a PCB with a humidity and temperature sensor (SHT25) and was compared to a DermaLab commercial device. Further testing in uncontrolled environments is needed to address factors such as air movement and humidity, which may affect the evaporation rate.

Bandodkar et al [29] introduced a wearable sweat-sensing platform combining battery-free, wireless electronic detection with integrated colorimetric assays (Figure S6 in Multimedia Appendix 1 [16,27-30]).

Lapadula et al [79] presented a system for analyzing body hydration to enhance running performance. It combined BIA as a benchmark with data from a Garmin Vivoactive device and a custom mobile app. The Garmin device recorded parameters such as HR, speed, altitude, calories, distance, and steps, whereas saliva analysis using cyclic voltammetry was used to extract hydration-related features. Challenges include validating the measurements and integrating these technologies into user-friendly wearable hydration monitoring for athletic activities.

Kamran et al [23] developed a data-driven method to measure hydration status using wearable sensors and normal orthostatic movements. Logistic regression models were trained to estimate dehydration status based on HR responses to postural movements, showing that shorter orthostatic tests achieved comparable accuracy to clinical tests. The study suggests that the sensor can accurately estimate mild dehydration in athletes. Challenges include expanding the range of movements and addressing study limitations such as the controlled exercise environment and specific postural movements used.

Sabry et al [80] used machine learning for on-device dehydration monitoring by integrating data from various wearable sensors such as accelerometers, magnetometers, gyroscopes, GSR, photoplethysmography, temperature, and barometric pressure. The focus on developing models suitable for memory-constrained wearable devices highlights their practical potential. Challenges include ensuring model accuracy while minimizing power consumption.

Rodin et al [81] assessed the real-world performance of a wearable body hydration sensor that integrates photoplethysmography and galvanic biosensors. The study involved 240 participants performing treadmill exercises over 90 minutes with intermittent rest periods to evaluate the sensor’s accuracy in monitoring water mass loss due to perspiration. The sensor, attached to a smartwatch, showed strong agreement with the gold-standard method (body mass change measured using a medical balance). The system uses proprietary algorithms to estimate sweat volume and a galvanic contact system to estimate total BWL, showing promising potential as an accurate wearable hydration monitor in practical real-world scenarios.

Wang et al [82] conducted a study predicting hydration status based on single-participant experiments involving 32 moderate-intensity exercise sessions with and without fluid intake. A total of 4 noninvasive physiological and sweat biomarkers—HR, core temperature, sweat sodium concentration, and whole-body sweat rate—were measured during exercise. Machine learning models were used to determine BWL percentage as an indicator of dehydration. The models revealed that whole-body sweat rate and HR achieved the highest accuracy, with sweat sodium concentration from the arms showing the best prediction accuracy. Challenges include understanding hydration’s impact on biomarker relationships with BWL percentage and highlight the need for future extended studies with multiple participants and intensity variations.

### Commercial Products and Comparisons

#### Overview

This section explores innovative hydration-monitoring solutions from leading companies. These products use advanced technologies, offering personalized insights through wearable patches, wristbands, and electronic biosensors. Table 4 provides an overview of various hydration-monitoring products, each with unique features and technologies.

**Table 4:** Overview of wearable hydration-monitoring system products.

Category	Name	Features	Validated	Commercial	Company	Technology
Wristband	Bioptx [83]	Hydration, temperature, HR^a^, HR variability, respiratory rate, and blood saturation monitoring	No	No	Rockley	IR^b^ laser technology (36 wavelengths), PPG^c^ (green, red, and IR), and accelerometer
Wristband	Sixty [84]	Hydration, HR, activity levels, calories burned, and sleep tracking	No	No	Sixty	Optical spectrometry; 3 LEDs shine green, red, and IR light
Wristband	LVL [85]	Hydration, activity, sleep, mood, HR, and calorie monitoring	No	No	BSX Athletics	Red light technology
Wearable band	hDrop Gen 2 [86]	Hydration and body temperature monitoring	No	Preorder	hDrop Technologies	Electrode tracks sweat loss and rate and sodium and potassium levels
Smartwatch	Geca sensor [87]	Hydration monitoring	Yes	Preorder	Hydrostasis	Optical spectroscopy and detection of fluid concentration in the skin
Smartwatch	Aura Strap [88]	HR, blood saturation, and hydration monitoring	No	No	Apple	Electrode measures electrolytes in sweat to monitor hydration
Smartwatch	Gx Sweat Patch [89]	Hydration, sweat, electrolyte content, and body temperature monitoring	Yes	Yes	Epicore Biosystems	A thin microfluidic substrate on the skin that captures sweat
Smartwatch	Hydration Biosensor [90]	Hydration monitoring	Yes	Yes	Nix	Electrochemical biosensors use electrodes to detect biomarkers in sweat [90]

^a^HR: heart rate.

^b^IR: infrared.

^c^PPG: photoplethysmography.

#### Rockley Bioptx

Bioptx is a noninvasive hydration-monitoring wristband using silicon photonics-based sensors (Figure S7 in Multimedia Appendix 1), which detect changes in water concentration in the body. Rockley’s platform is aimed at athletes, health-conscious individuals, and those managing hydration-related conditions [91].

#### Sixty

Sixty offers a hydration-monitoring solution with simple design and LED indicators. Users can choose between continuous monitoring using a wrist or arm strap or occasional checks using the stand-alone monitor. This flexibility caters to both athletes needing constant monitoring and individuals seeking occasional hydration updates. The device can detect fluid loss as low as 30 to 40 mL (approximately 1.35 oz), providing timely reminders before the sensation of thirst, typically felt after losing 300 to 400 mL (approximately 13.53 oz) of fluid. Sixty plans to gather extensive data to ensure accuracy across different phenotypes [91,92] (Figure S8 in Multimedia Appendix 1 [16,27-30]).

#### LVL Wristband

The LVL wearable hydration monitor uses red light technology, which penetrates deeper into the body than conventional fitness trackers that use green light sensors. Designed for individuals seeking comprehensive health monitoring, LVL calculates fluid requirements based on hydration status and sweat rates. The device emphasizes the connection between hydration and improved sleep quality and offers compatibility with various devices through BLE technology [85] (Figure S9 in Multimedia Appendix 1 [16,27-30]).

#### hDrop Gen 2

hDrop Gen 2 from hDrop Technologies provides rapid, direct skin contact readings for sweat rate, electrolyte concentration, and estimated sodium and potassium levels. Primarily aimed at athletes, this technology uses electrodes to offer personalized recommendations for fluid and electrolyte replenishment, improving hydration management during workouts [93] (Figure S10 in Multimedia Appendix 1 [16,27-30]).

#### Hydrostasis Geca Sensor

The Geca sensor is an optical-based wristband that underwent rigorous evaluation, including comparisons with known hydration and dehydration events and urine-specific gravity. Consumer pilot studies show the sensor’s potential to accurately measure hydration changes. It demonstrates greater sensitivity in predicting hydration levels associated with minimal total body weight changes ranging from 0.5% to 1%, outperforming conventional salivary osmolality tests and standard physical assessments of dehydration [94] (Figure S11 in Multimedia Appendix 1 [16,27-30]).

#### Aura Strap

The Aura Strap integrates smart functionality into an Apple Watch band to track hydration levels using BIA. It also provides insights into body composition, including body fat, muscle mass, minerals, and body water, when paired with user information such as age, gender, and weight. Measurements are taken by placing the sensor on a specific part of the hand, with data transmitted to the Apple Watch and stored in the dedicated app. However, it only offers spot measurements rather than continuous monitoring, and improvements in size and ease of use are needed [95].

#### Epicore Gx Sweat Patch

Epicore Biosystems’ Gx Sweat Patch is a wearable technology aimed at athletes seeking personalized hydration insights and recovery guidance. The skinlike patch pairs with the Gatorade Gx iOS app, using microchannels to capture sweat data in real time. The app provides athletes with personalized recommendations based on their sweat profiles, catering to their unique hydration and refueling needs. A paper was published detailing their wearable microfluidic-based system for real-time analysis of sweat rate and sweat chloride concentration, validated through studies involving 312 athletes [89,96] (Figure S13 in Multimedia Appendix 1 [16,27-30]).

#### Nix Hydration Biosensor

The Nix Hydration Biosensor is a lightweight, compact electronic device designed for durability and reusability. It attaches to an individual sweat patch and continuously transmits data to a smartphone, watch, or bicycle computer, with a battery life of up to 36 hours, making it suitable for long training sessions. The Nix app offers real-time data for monitoring fluid and electrolyte loss, historical data to understand sweat profiles, and predictive data that calculate future hydration needs based on weather forecasts and past performance [90].

## Discussion

### Principal Findings

#### Overview

The studies revealed that many wearable devices are either in the prototype phase or tailored to specific user groups. Table 5 provides a concise overview of the distribution of publications across various hydration-tracking techniques, categorizing them into electrical, optical, thermal, microwave, and multimodal methods. This categorization sheds light on the current landscape of research in the field [83].

**Table 5:** Number of publications by sensor category (N=63).

Sensor category	Publications, n (%)
Electrical	31 (49)
Optical	13 (21)
Thermal	4 (6)
Microwave	7 (11)
Multimodal	8 (13)

#### Electrical-Based Sensors

Electrical-based methods, particularly BIA, are currently leading the field, with 49% (31/63) of the publications underscoring their widespread use and popularity. These methods are favored because they directly correlate the skin’s electrical properties (such as impedance, capacitance, and conductance) with hydration levels. This relationship allows for the efficient measurement of hydration states through noninvasive, rapid, and quantitative assessment. In addition, BIA systems stand out due to several practical advantages, including their accuracy, user-friendliness, portability, and affordability. These characteristics make them particularly suitable for applications in dermatology, cosmetics, and material testing, as well as for clinical and field-based studies requiring real-time monitoring [17,68].

Despite these advantages, electrical-based methods, including BIA, face significant limitations. One major drawback is their limited ability to assess hydration in deeper dermal layers. While these methods excel at evaluating hydration within the outermost layer, the stratum corneum, they may not provide comprehensive insights into fluid distribution deeper in the skin, such as in the epidermis or dermis. This limitation can be critical in contexts in which deeper hydration dynamics are integral to understanding skin health or the effectiveness of topical treatments. In addition, these systems are highly sensitive to external factors, including environmental conditions [97] (eg, humidity and temperature) and physiological influences (eg, skin temperature, sweat, and lipid content). For instance, higher ambient humidity or temperature can artificially increase skin conductance by softening the stratum corneum or inducing sweating, leading to overestimated hydration levels. In fluctuating or extreme environmental conditions, these sensitivities may compromise the accuracy and reliability of hydration measurements unless properly controlled or calibrated [98].

Efforts have been directed at refining BIA for more robust performance, such as using multi-frequency impedance measurements to capture both extracellular and total water content, integrating environmental monitoring and machine learning for calibration, and advancing hybrid systems that combine BIA with complementary methods. These innovations aim to mitigate environmental and depth measurement limitations while enhancing the method’s sensitivity and validity. As a result, BIA remains a leading focus in skin hydration assessment research, offering high utility and adaptability and the potential for further performance optimization in diverse settings [17,68,69].

Electrochemical methods, which monitor hydration by measuring sodium ion and potassium ion levels, offer higher accuracy due to their specificity in monitoring electrolyte levels. However, they face challenges such as ion selectivity issues and electrode conditioning problems [99]. In addition, variability in fluid extraction processes across individuals adds complexity to achieving consistent measurements.

Each subcategory of electrical-based sensors offers distinct advantages and limitations. Electrical sensors are ideal for quick, noninvasive assessments, particularly in sports and fitness settings in which surface hydration measurements suffice. In contrast, electrochemical sensors are more suited for applications requiring precise electrolyte monitoring, such as medical diagnostics and sports science.

#### Optical-Based Sensors

Optical-based hydration tracking techniques were assessed in 21% (13/63) of the publications. While not as widespread as electrical methods, they provide distinct advantages, particularly their ability to deliver detailed molecular information about skin hydration. The smaller number of publications in this area may be due to the complexity and cost of advanced optical sensors. Recent advancements in LED-based and semiconductor photonic sensors have addressed some of these challenges by offering smaller sizes and lower costs than traditional laser-based laboratory equipment. These developments in semiconductor miniaturization and photonics technology present promising opportunities for hydration monitoring.

While infrared spectroscopy and NIRS focus on water absorption at key wavelengths (notably, 1300 nm for maximum skin penetration and 1480 nm for sensitivity to water concentrations), some studies (3/63, 5%) extended this range to include 940 nm to encompass sweat properties. Additional frequencies can lead to improved accuracy. However, the challenge remains in refining sensor sensitivity and specificity. Achieving consistent accuracy across different skin types, conditions, and environments is a significant hurdle, and variability in individual skin properties and external factors such as lighting conditions can impact measurement reliability [100].

Current optical methods primarily assess surface or near-surface hydration levels, limiting their ability to capture deeper skin layers or the body’s overall hydration status. Developing techniques with deeper penetration capabilities while ensuring safety and ease of use could enhance the effectiveness of optical hydration tracking.

Leveraging AI and machine learning algorithms to analyze the vast amounts of data collected by these sensors could further improve their accuracy, sensitivity, and ability to adapt to individual variations and environmental changes. Optical sensors are particularly well suited for applications requiring detailed surface hydration analysis, such as skincare and cosmetics, where precision is paramount.

#### Thermal and Microwave-Based Sensors

Other methods such as thermal and microwave-based sensors were identified, comprising 17% (11/63) of the publications. While these methods represent a smaller portion of the research landscape, they cater to specific applications and niches. Thermal sensors offer a unique perspective on skin hydration, probing the skin’s thermal properties for insights. They have seen notable advancements, particularly in the development of sensors capable of measuring thermal properties beneath the skin’s surface. In addition, some sensors measure skin temperature and thermal transport for evaluating skin hydration and blood flow dynamics. While promising, thermal sensors face challenges in terms of precision and sensitivity. Calibration is crucial, and factors such as ambient temperature can influence the results.

Microwave-based systems leverage electromagnetic waves’ interaction with water molecules to assess skin moisture. These sensors are noninvasive and can penetrate deeper layers of the skin. However, such systems face several challenges that could limit their practical application. One of the main drawbacks is the difficulty in miniaturizing these systems to fit comfortably and unobtrusively into wearable devices. The complexity and size of the components required for microwave sensing can make it challenging to develop compact, wearable devices that are convenient for everyday use. Another significant limitation is the power consumption of microwave sensors. High power requirements can lead to shorter battery life in wearable devices, reducing their practicality for continuous, long-term monitoring In addition, microwave sensors can be susceptible to interference from external electromagnetic sources, which can affect the accuracy and reliability of hydration measurements. Future improvement opportunities in this field should look to achieve greater miniaturization and integration into wearable devices, addressing power consumption issues and making continuous hydration monitoring more feasible. Furthermore, developing advanced algorithms for signal processing and interference rejection could enhance the accuracy and reliability of microwave-based hydration tracking even in environments with significant electromagnetic noise.

Thermal sensors are best suited for applications such as medical diagnostics, where detailed thermal assessments of hydration are needed. Microwave sensors are more appropriate for applications demanding deep tissue analysis, although practical implementation challenges persist.

#### Multimodal Sensors

A total of 13% (8/63) of the publications explored the use of multimodal sensors for hydration monitoring. This emerging trend suggests that researchers are recognizing the potential benefits of combining multiple sensor types to enhance accuracy and reliability. Multimodal sensors could address limitations associated with single-mode sensors, providing a more comprehensive understanding of hydration dynamics by addressing the limitations of individual sensors. As technology continues to advance, we can expect to see further exploration of this approach.

However, integrating multiple sensor technologies into a single wearable device introduces a set of challenges and limitations. One primary drawback is the complexity involved in sensor integration and the management of data from diverse sources. Ensuring that sensors operate harmoniously without interfering with each other’s signals requires careful design and calibration. In addition, the increased complexity might lead to higher power consumption, larger device size, and potentially higher costs, which could limit the practicality and accessibility of multimodal hydration monitors for everyday use.

Another significant challenge is optimizing data fusion algorithms to combine data from multiple sensors meaningfully. This is crucial for interpreting complex data streams but requires algorithms that can accurately process relevant data from each sensor considering context and potential errors or noise.

Multimodal sensors are suitable for applications requiring comprehensive hydration analysis, such as health care and elite sports, where precise monitoring is crucial.

#### Commercial Products and Practical Implementations

Our analysis of commercial hydration-monitoring products revealed a growing market with a diverse range of offerings. Companies are effectively translating research into practical products for consumers, athletes, and health care practitioners. Products such as the Gx Sweat Patch and Nix Hydration Biosensor demonstrate the potential for providing actionable insights into hydration management. Optical-based techniques, which are cost-effective and practical for real-time monitoring, are gaining traction in the market due to their versatility and convenience.

While research provides the foundation for hydration-monitoring technologies, translating findings into commercially viable products presents several challenges. Controlled research settings often differ significantly from real-world conditions, where factors such as skin properties, sweat composition, and environmental variations can impact sensor accuracy. In addition, commercial products must balance accuracy with user convenience, power efficiency, and cost-effectiveness to ensure practicality in everyday use. Regulatory approval processes such as FDA certification for medical-grade devices further influence how research findings can be integrated into consumer products. The ability to refine prototype technologies into scalable, market-ready solutions depends on advancements in sensor miniaturization, robust validation across diverse populations, and improved data interpretation algorithms to enhance reliability under variable conditions

Ensuring the successful transfer of research advancements into commercial hydration-monitoring solutions requires overcoming key technical and practical challenges. Future efforts should focus on optimizing sensor performance in diverse real-world settings, improving long-term stability and accuracy, and addressing user adoption factors such as device comfort and accessibility. In addition, integrating hydration monitoring into multifunctional wearable systems—such as smartwatches and fitness trackers—can enhance usability and increase adoption. Continued refinement of these technologies will determine their effectiveness and scalability, ultimately shaping the future of hydration-monitoring solutions in both consumer and professional markets.

### Limitations

This scoping review has some limitations. The inclusion of studies with varying validation methods makes direct comparisons challenging as methodological differences obstruct the establishment of standardized benchmarks for device accuracy and reliability. In addition, the lack of long-term evaluations limits insights into the real-world applicability of both academic research findings and commercial products. While the commercial product search provides practical insights into market trends and user-centered innovations, reliance on gray literature introduces potential bias. Many commercial products, including some mentioned in this review, lack rigorous scientific validation, raising concerns about the reliability and reproducibility of their claims [85,86,88]. Finally, the limited number of large-scale clinical trials addressing device effectiveness significantly restricts the ability to draw robust conclusions about the applicability of these technologies across diverse populations. This is one of the most significant criticisms of current hydration tracking research as small-scale studies and laboratory-controlled settings fail to capture variations in hydration status markers caused by differences in age, physiology, activity levels, environmental conditions, and health status. Addressing these gaps through standardized validation protocols, long-term real-world testing, and scaled clinical trials is essential to strengthen confidence in these technologies both for research advancement and consumer trust in emerging products.

### Conclusions

This scoping review provides a comprehensive analysis of recent advancements in wearable hydration-monitoring technologies, offering insights into their capabilities, limitations, and research trends. This review highlights the growing interest in noninvasive, portable hydration-monitoring systems using physiological biomarkers, with a predominant focus on electrical-based sensors such as BIA due to their ease of use and accuracy. Optical-based sensors, while less common, offer molecular-level insights and are gaining traction with advancements in photonic technology. Thermal and microwave-based sensors cater to specific applications but face challenges regarding precision and miniaturization. The emergence of multimodal sensors suggests a growing trend toward combining technologies for enhanced accuracy and reliability. Commercially available hydration-monitoring products demonstrate a growing market with diverse offerings, primarily dominated by optical-based solutions due to their cost-effectiveness and the diverse number of biomarkers that they give access to.

Future research should focus on enhancing sensor accuracy, miniaturization, and wearability; standardizing measurement protocols; and refining data interpretation algorithms. Multimodal systems and AI-driven analysis show promise for personalized hydration management, with broad applications in health care and beyond. Overall, wearable hydration-monitoring devices offer significant potential for real-time hydration assessment. Overcoming challenges such as reliability, accuracy, and applicability across diverse populations will be crucial for their widespread adoption and impact in health care and performance optimization.

## Appendix

Multimedia Appendix 1 Supplementary figures illustrating key experimental setups and device schematics referenced in this review.

Multimedia Appendix 2 PRISMA-ScR (Preferred Reporting Items for Systematic Reviews and Meta-Analyses extension for Scoping Reviews) checklist.

## Abbreviations

AI: artificial intelligence
BIA: bioelectrical impedance analysis
BLE: Bluetooth Low Energy
BWL: body water loss
FDA: Food and Drug Administration
GSR: galvanic skin response
HR: heart rate
MCU: microcontroller unit
NFC: near-field communication
NIRS: near-infrared spectroscopy
PCB: printed circuit board
PRISMA: Preferred Reporting Items for Systematic Reviews and Meta-Analyses
PRISMA-ScR: Preferred Reporting Items for Systematic Reviews and Meta-Analyses extension for Scoping Reviews
SHS: skin hydration sensor
smLED: surface-mount light-emitting diode
TDR: time-domain reflectometry
