# Supplementary material for: Recent Advancements in Wearable Hydration-Monitoring Technologies: Scoping Review of Sensors, Trends, and Future Directions
Source: JMIR Mhealth Uhealth. 2025 Jun 13;13:e60569. doi: 10.2196/60569 (PMC12205265; doi:10.2196/60569)
Supplement: Multimedia Appendix 1 [file mhealth_v13i1e60569_app1.docx]

**Supplementary Documents**


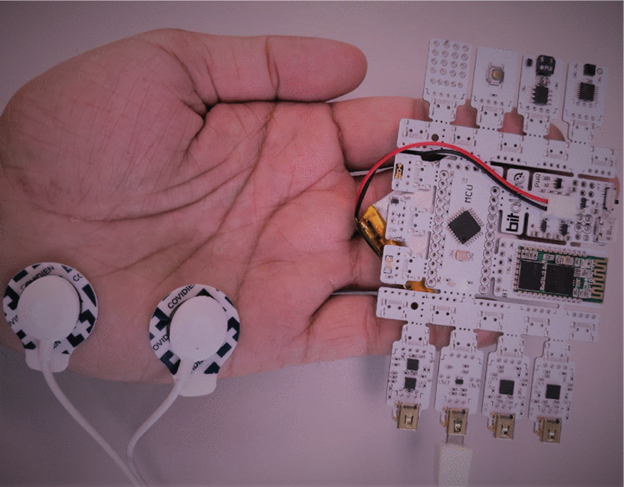

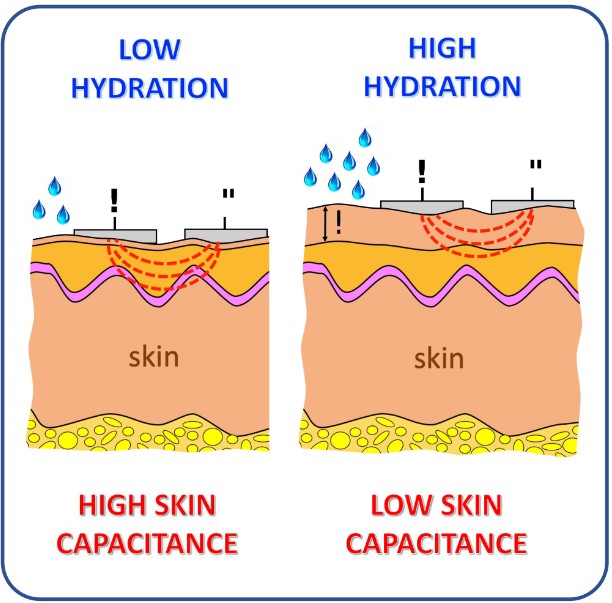


Figure 4:(supplement) The correlation between skin capacitance and hydration [99].

Figure 5: (supplement) GSR data collection using BITalino EDA sensor by placing two electrodes [16].


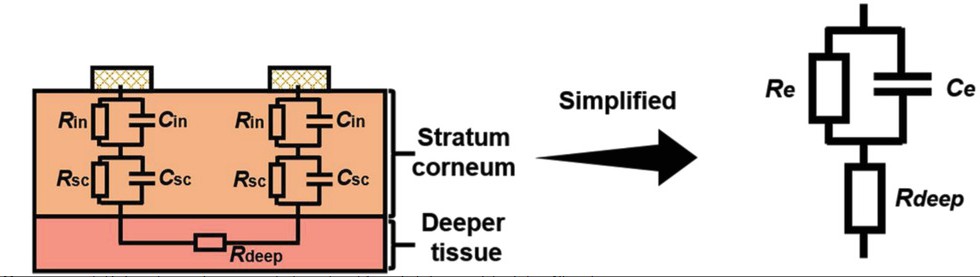


Figure 6:(supplement) Skin impedance analysis simplified model [41].


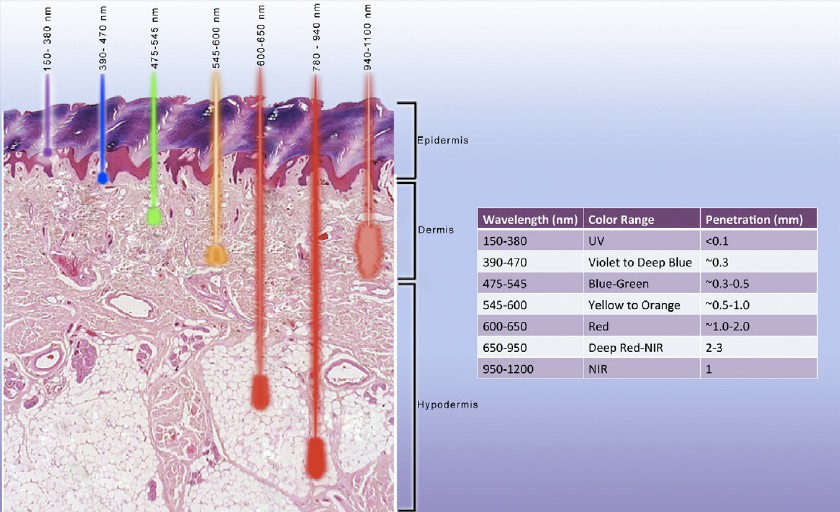


Figure 7:: (supplement) Tissue penetration depths of various wavelengths. (Figure courtesy of Well- man Center for Photomedicine.)


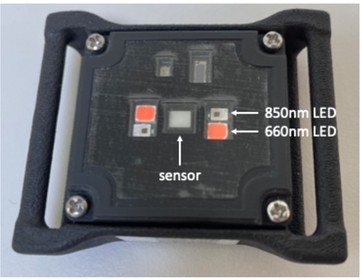


Figure 8:(supplement) Wearable Compact CMOS spectrometer for skin hydration monitoring [61].


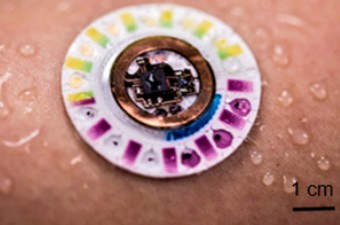


Figure 9:(supplement) Microfluidic patch with embedded sensors operation during sweat [77]


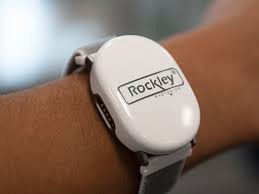

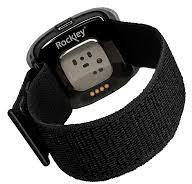


Figure 10:(supplement) Bioptx™ Biosensing Band


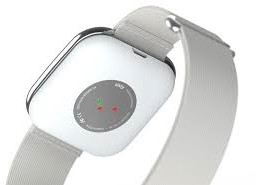

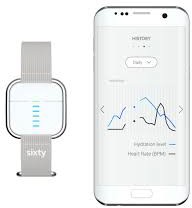


Figure 11: (supplement) Sixty App-Enabled Hydration Monitor


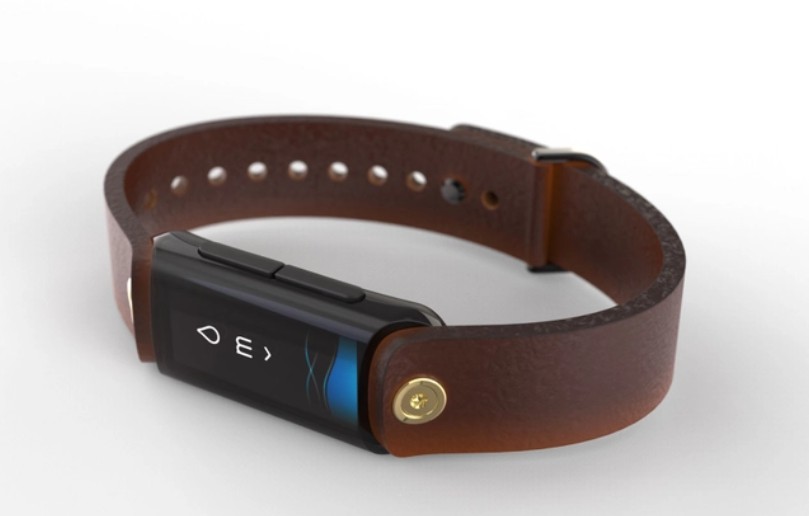

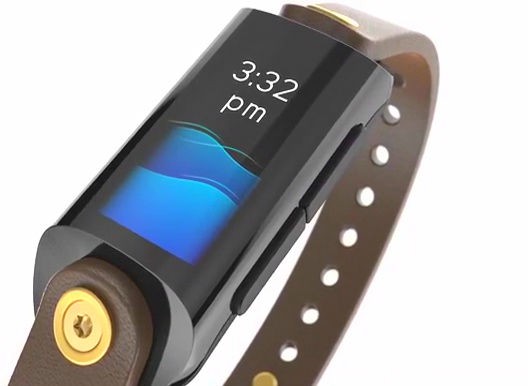


Figure 12:(supplement) LVL wearable hydration monitor


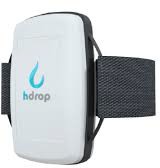


Figure 13: (supplement) hDrop Gen 2 - wearable hydration sensor


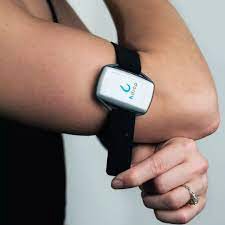


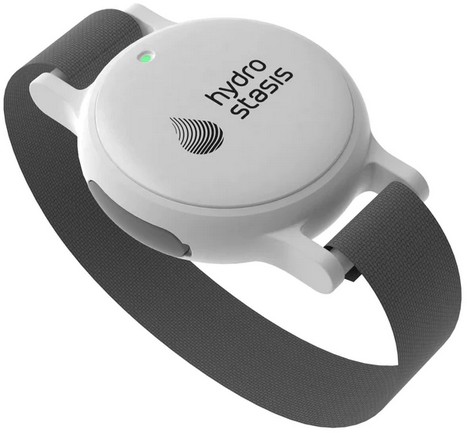

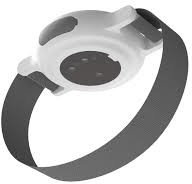


Figure 14:(supplement) GECA™ Hydration Watch


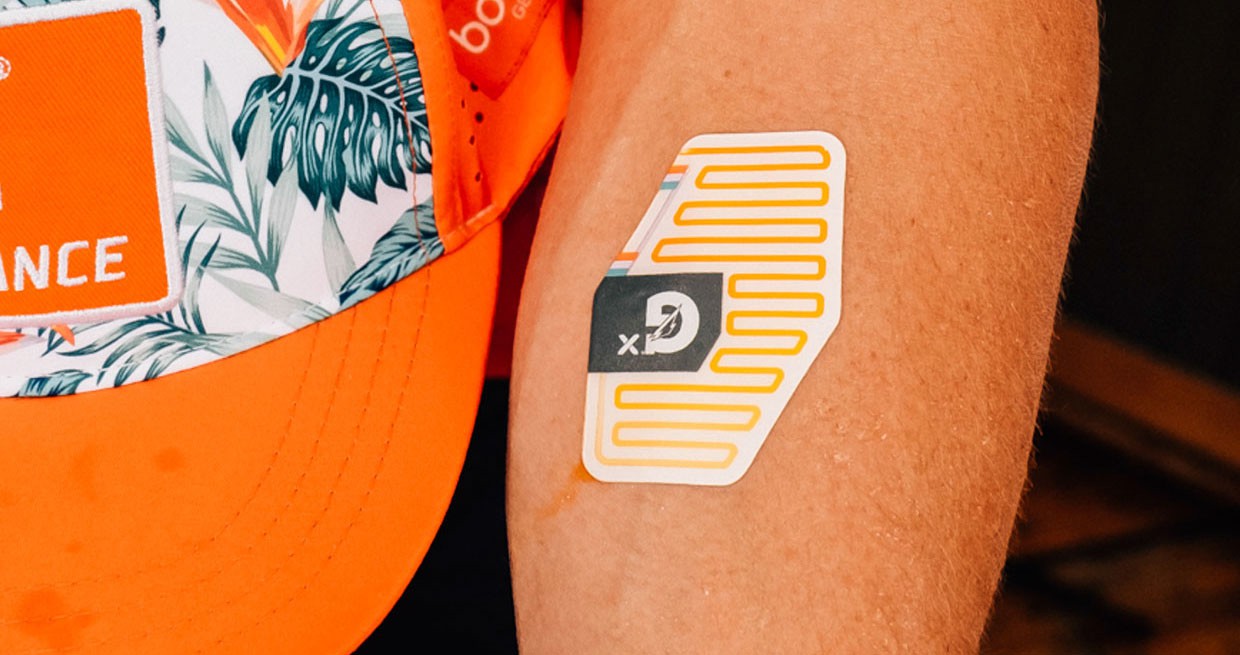

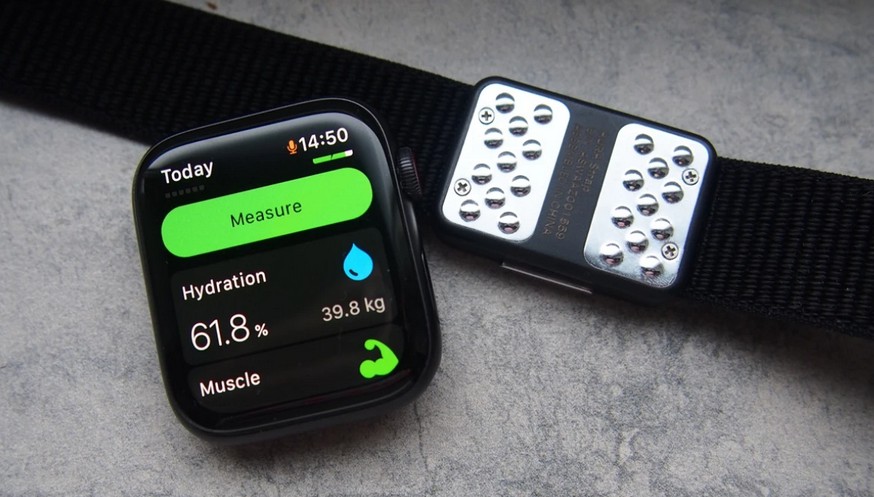


Figure 15:(supplement) AURA Strap 2

Figure 16:(supplement) Gx Sweat Patch


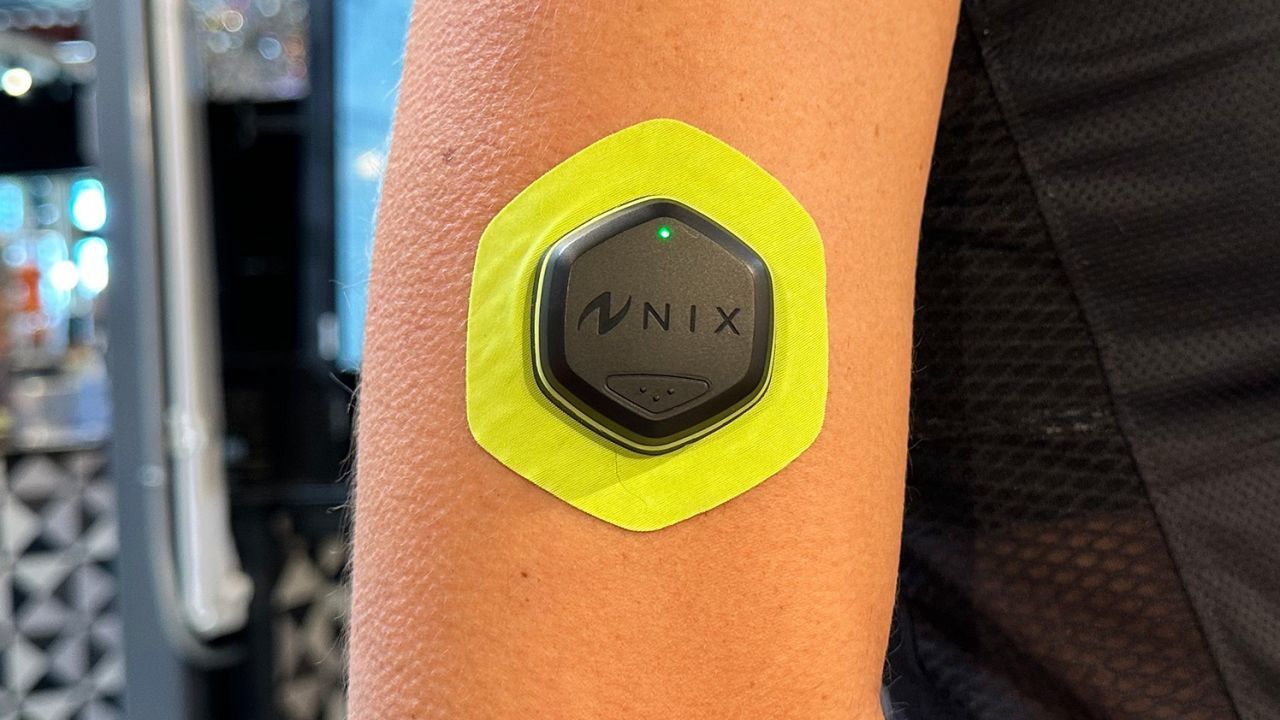


Figure 17:(supplement) Nix Hydration Biosensor Patch.
